# Explaining how mutations affect AlphaFold predictions

**DOI:** 10.64898/2025.12.30.697132

**Published:** 2025-12-31

**Authors:** Madeleine F. Clore, Joseph F. Thole, Suchetan Dontha, Pramesh Sharma, Davin Jensen, Brian F. Volkman, Matthew Coudron, Lauren L. Porter

**Affiliations:** 1. National Library of Medicine, National Institutes of Health, Bethesda MD 20894, USA; 2. National Heart Lung and Blood Institute, National Institutes of Health, Bethesda MD, 20894, USA; 3. The Joint Center for Quantum Information and Computer Science, University of Maryland, College Park, MD 20742; 4. Department of Biochemistry, Medical College of Wisconsin, Milwaukee, WI 53226, USA

## Abstract

Transformer models, neural networks that learn context by identifying relationships in sequential data, underpin many recent advances in artificial intelligence. Nevertheless, their inner workings are difficult to explain. Here, we find that a transformer model within the AlphaFold architecture uses simple, sparse patterns of amino acids to select protein conformations. To identify these patterns, we developed a straightforward algorithm called Conformational Attention Analysis Tool (CAAT). CAAT identifies amino acid positions that affect AlphaFold’s predictions substantially when modified. These effects are corroborated by experiments in several cases. By contrast, modifying amino acids ignored by CAAT affects AlphaFold predictions less, regardless of experimental ground truth. Our results demonstrate that CAAT successfully identifies the positions of some amino acids important for protein structure, narrowing the search space required to make effective mutations and suggesting a framework that can be applied to other transformer-based neural networks.

## Introduction

Artificial intelligence methods have been applied with great success to many challenging problems including text and image generation (1, 2), diagnostic imaging in healthcare (3), and predicting protein structures and interactions (4–6). Many of these successful models are based on transformer architectures (7), which can readily identify deep, context-dependent relationships in sequential data. Nevertheless, the opaque inner workings of these AI models make it difficult to understand the mechanistic basis for their failures (8). We sought to understand how the most successful AI models for protein structure prediction–AlphaFold2 (AF2) (6) and AlphaFold3 (AF3) (5)–select between different conformations of fold-switching proteins. Fold switchers remodel their secondary and/or tertiary structures and modulate their functions in response to cellular stimuli (9), offering clearly distinguishable benchmarks for prediction assessment and addressing why predicting diverse protein conformations remains a challenge (10). Leveraging fold switchers as adversarial examples for AF2 and AF3 (AF) (11), we aimed to identify sequence features that AF uses to predict a specific protein conformation.

## AlphaFold uses simple patterns to select XCL1 conformations

**Figure 1.**
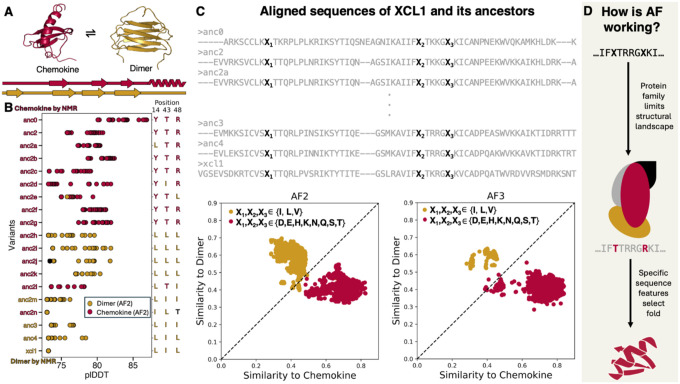
AlphaFold selects XCL1 conformations based on the identities of three amino acids. A. XCL1 is a human immunoprotein that interconverts between chemokine (red) and dimer (gold) conformations with completely different tertiary structures and hydrogen bonding networks. B. AF2 overpredicts the dimer conformation (gold circles) among XCL1 variants. Red names were observed to assume the chemokine fold only; gold names exchanged between dimer and chemokine. Anc2e, 2h, 2i, 2j, and 2k are all predicted to assume the dimer conformation, which was not detected experimentally. Positions 14, 43, and 48 of these proteins’ aligned sequences are enriched in Y, T, R, respectively, for the chemokine conformation, and L, I for the dimer conformation. C. AF2 and AF3’s conformational selection process for XCL1 can be reduced to simple patterns based on the identities of three amino acids (black Xs). The dimer fold (gold circles) was predicted for nearly all combinations of X in {I,L,V}, while the chemokine fold (red circles) was predicted for many combinations of X in {D,E,H,K,N,Q,S,T}. Identities of all gray amino acids were fixed. D. AF is hypothesized to work as follows. The overall input sequence (protein family) limits the structural landscape, that is the specific conformations that AF predicts. Then specific sequence features select a fold from that landscape.

We first tested AF on the human immunoprotein XCL1, which exchanges between a monomeric chemokine and a dimeric β-sheet fold (12) (Figure 1A). Each conformation performs a distinct function: GPCR signaling (chemokine) and fungal pathogen binding (dimer) (13). Dishman *et al*. characterized XCL1, four reconstructed ancestors (Anc0,2,3,4), and fourteen variants of Anc2 (a-n) to pinpoint specific physical constraints enabling conformational exchange (14). Nuclear magnetic resonance (NMR) experiments indicated that 15/19 of these variants assume the monomeric chemokine fold only, while the remaining 4 (anc2m, anc3, anc4 and XCL1) exchange between both conformations (14). To test how AF predictions correspond to experiments, we ran AF in single sequence mode on all 19 variants. Previous work has shown AF can distinguish between single-folding and fold-switching chemokines from single sequences but not full multiple sequence alignments (11). Further, single sequences allow analysis of how AF predicts protein structures without an input multiple sequence alignment (MSA), which can bias predictions (15, 16).

AF overpredicted the dimer conformation substantially. Though observed in 4/19 variants, it was predicted with good confidence in 10/19 variants by both AF2 and AF3 (Figure 1B, S1). To determine which specific amino acids inform AF’s predictions, we identified 42 chemokine PDB structures deposited prior to AF training and two dimer structures (Figure S2A). Individual substitutions in sequence positions that differed most between dimer and chemokine structures were performed (Figure S2B, Methods). Though most individual substitutions did not change the conformation predicted, position 48 led to changes in both directions (Figure S3). Further, all chemokine predictions had a T in position 43 or an R in position 48, whereas dimer predictions were enriched for I and L in these positions as well as position 14 (Figure 1B, S1).

For XCL1 and its experimentally characterized variants, AF predicted the dimer and chemokine conformations based on simple patterns of amino acids in positions 14, 43 and 48 (Figures 1C). Specifically, variants with nearly all combinations of {I,L,V} in these positions were predicted to assume the dimer conformation, whereas most combinations of {D,E,H,K,N,Q,S,T} were predicted chemokine (Methods). Thus, AF relies on simple patterns to select between chemokine and dimer folds. Importantly, however, the identity of these three amino acids is not the only factor required. AF does not select between XCL1 conformations from polyalanine chains with {I,L,V} and {D,E,H,K,N,Q,S,T} in positions 14, 43, and 48. Instead, we hypothesize that the sequence background of the XCL1 family narrows the number of possible conformations AF predicts, and the identities of the three amino acids in positions 14, 43, and 48 play an important role in selecting the overall conformation (Figure 1D). Interestingly, mutating the same positions of a more distant homolog of XCL1––MCP-2––induced the same conformational change, indicating that AlphaFold sometimes mistakenly applies this simple pattern to other chemokine clades in which the dimer conformation has not been observed (Figure S4).

## AF’s selection points for XCL1 correspond to amino acids with high attention

**Figure 2.**
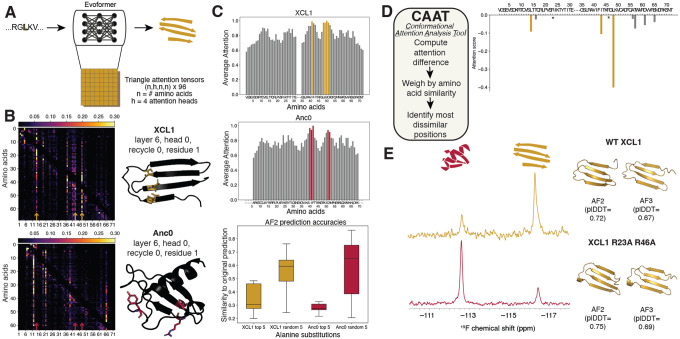
Amino acids important for XCL1 folding and conformational selection can be identified readily from AF2’s triangle attention tensors. A. The basic approach: a sequence is inputted into ColabFold, triangle attention heads are extracted from its Evoformer block, and three-dimensional structures are predicted. B. Triangle attention heads indicate high attention is paid to the three amino acids that AF uses to select the dimer fold (columns with gold arrows). These three positions correspond to an interaction network specific to XCL1’s dimer interface (yellow amino acids on black dimer structure). The same positions receive less attention when the chemokine structure of Anc0 is predicted (columns with red arrows). In this conformation, these three amino acids do not form an interaction network (red amino acids on black chemokine structure). C. Positions with high attention inform AF2 predictions of both folds. Rescaled attention values for the dimer (top) and chemokine (middle) plots and their top 5 most attended residues (gold and red, respectively). Mutating these top 5 positions to alanine decreases prediction accuracy significantly more than alanine mutagenesis of 5 random positions (bottom). D. To identify amino acids important for selecting a given fold, we took the difference between the XCL1 and Anc0 plots in C. These differences were weighted by amino acid similarity, since more different amino acids would be expected to switch predictions. This approach is called CAAT (Conformational Attention Analysis Tool). The resulting difference plot yielded the three amino acids uniquely important for dimer fold predictions. E. Mutating positions with low attention (starred positions in D) has little effect on predictions, though it has a large effect experimentally. XCL1 is observed to populate 80% dimer/20% chemokine by ^19^F NMR, while XCL1 R23A R43A populates 20% dimer/80% chemokine. R23A and R43A receive relatively little attention from the AF2 network (Figures 2C,D).

Because both AF2 and AF3 used similar simple patterns to select XCL1 conformations (Figure 1C), we hypothesized that a common element of their network architectures may be the cause. In both networks, three-dimensional structures are generated based on pairwise restraints inferred from amino acid sequence using a transformer model. In this case, the model relates a given amino acid to every other amino acid in the protein sequence through a mechanism called attention (7). Amino acid pairs with strong relationships are expected to be close in space. Thus, we hypothesized that XCL1 variants with {I,L,V} in positions 14, 43, and 48 cause AF to produce different attention patterns–and hence different folds–than the chemokine-producing amino acids in those positions. To test this hypothesis, we extracted the triangle attention heads– tensors used to identify pairwise relationships between amino acids–of XCL1 and its variants from the AF2 network (Figure 2A). We focused on AF2 because both its code and weights are fully open source, allowing us to release resulting software to the public with no restrictions. For ease, we used an efficient but accurate implementation of AF2 called ColabFold (17).

XCL1 attention heads displayed an interaction network unique to the dimer fold (Figure 2B). Using an interpretation strategy originally suggested by the AF team (6), this network is characterized by vertical lines corresponding to interacting amino acids (Figure S5A,B). Importantly, this network is not present in the corresponding attention heads of Anc0, which AF correctly predicts chemokine (Figure 2B, S5C). Further, two Anc2f sequences differing only in positions 14, 43, and 48 and predicted as dimer (LLL) or chemokine (YTR) displayed similar attention patterns (Figure S6). These results indicate that AF2 selects XCL1 conformations based on sequence patterns unique to the dimer conformation; AF3 has likely learned similar patterns since it produces similar predictions (Figures 1C, S1). Although these patterns are not sufficient to engender an experimentally detectable population of the dimer conformation (14), they are common features among XCL1 sequences that form experimentally characterized dimers (Figure 1B).

Extending this approach, we averaged the attention over each residue column across all attention heads from all five AF2 models and rescaled the values for ease of interpretation (Methods). While positions 14, 43, and 48 had scaled attention values between 0.6 and 0.9 for XCL1 (dimer), corresponding values in Anc0 (chemokine) ranged from 0.4–0.6, again highlighting the elevated importance of these three positions for dimer predictions (Figure 2C). Consistent with our hypothesis that AF uses overall sequence background to limit number of possible structures predicted, 3/5 most attended amino acids were in positions common to both folds. To test our hypothesis, we mutated the top 5 most attended amino acids for both XCL1 and Anc0 to alanine and generated 100 AF2 models for each mutated sequence. Consistent with our hypothesis, the accuracies of these mutated models were significantly lower than predictions of sequences with alanine mutations in 5 random positions (Figure 2C). These results indicate that highly attended amino acids inform AF2 predictions more than others, potentially explaining why AF fails to predict the effects of some mutations (18, 19).

To identify amino acids uniquely important to each fold, we took differences between the attention values corresponding to the different folds and scaled each value by their amino acid similarities. We expected that amino acids receiving high attention, and whose chemical properties differ from those in the alternative fold, would be most likely to cause AlphaFold to switch its prediction. For instance, the dimer fold favors aliphatic amino acids in position 48 whereas the chemokine fold favors charged and polar (Figures 1B, S2B). This strategy–which we call the Conformational Attention Analysis Tool (CAAT)–yielded positions 14, 43, and 48 as most important for the dimer conformation (Figure 2D). Applying a linear classifier to XCL1 sequences with random alanine mutations also highlighted the importance of two of these amino acids to the dimer fold, though this approach required much more sampling (Figure S7).

Predictions of another XCL1 variant with mutations to sites of lower attention were affected less than the uniquely important amino acids identified by CAAT. Specifically, while ^19^F-NMR experiments indicate that wildtype XCL1 populates the dimer fold 77% of the time, under the same conditions XCL1 R23A,R43A populates the dimer fold 26% of the time (Figure 2E). Inconsistent with experiment, AF2 and AF3 predict that XCL1 R23A,R43A assumes the dimer conformation with slightly higher confidence than wildtype. This may be because these positions receive relatively little attention in either fold.

## CAAT identifies important amino acids in three other fold-switching systems

**Figure 3.**
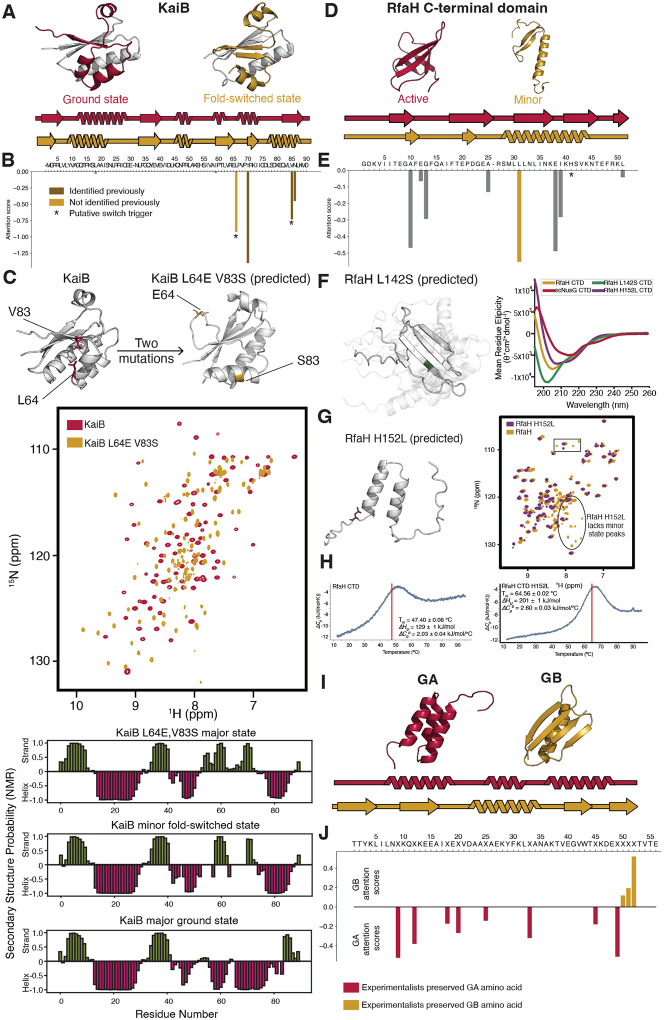
CAAT predictions confirmed by experiment in three additional fold-switching systems. A. The circadian clock protein, KaiB, interconverts between a major ground state (red) and a minor fold-switched state (gold). B. CAAT suggested that 4 amino acids were important to the ground state fold. Three had been identified previously. C. AF2 predictions suggested that mutations to the previously unidentified site (L64) and a second site (V83) were sufficient to switch KaiB’s fold. The ^1^H,^15^H heteronuclear single quantum coherence spectrum (HSQC) of KaiB shifts dramatically in response to these two mutations (red, wildtype; gold, double mutant). Backbone assignments indicate that the secondary structure of the double mutant’s major state matches the minor fold-switched state of wildtype. D. The C-terminal domain of transcription regulator RfaH interconverts between a major β-roll fold and a minor unfolded conformation with helical and b-sheet character. E. CAAT predictions indicate that L in position 142 is the most important for RfaH CTD’s fold, while H in position 152 receives no appreciable attention. F. AF2 predicts numerous low-confidence structures with distinct conformations in response to the L142S mutation. Its circular dichroism spectrum confirms that it is unfolded. G. AF2 confidently predicts that RfaH’s minor state is preserved in response to the low-attention H152L mutation. Its HSQC indicates the opposite: the H152L mutation wipes out many peaks corresponding to RfaH’s minor state. H. Consistent with the undetectable minor state, differential scanning calorimetry profiles indicate that the major state of RfaH H152L is more stable than wildtype, with melting temperatures of 64.6 and 47.4°C, respectively. I. The proteins GA and GB were engineered to switch folds in response to one mutation. J. Starting from variants of GA and GB with 77% sequence identity, high-attention positions identified by CAAT correspond exactly with amino acids that the GA/GB designers chose to preserve the GA and GB folds. X positions in sequence indicate those varied in subsequent designs.

CAAT’s success with XCL1 prompted us to test whether it extends to other known fold-switching proteins. We started first with KaiB, a critical component of bacterial circadian clocks (20). *Rhodobacter sphaeroides* KaiB (KaiBRs) populates an inactive ground state and a minor active fold-switched state (Figure 3A) that binds KaiC, another critical component of the bacterial circadian oscillator (21, 22). From single sequence, AF2 predicts KaiBRs to assume the ground state conformation, while it predicts the fold-switched conformation for *Legionella pneumophila* KaiB (KaiBLp). Using these two proteins as input, CAAT suggested 4 amino acids important to the ground state fold (Figure 3B). By comparing AF2 predictions of hundreds of sequences, three of these amino acids were identified and confirmed to switch KaiBRs’s fold (15). By contrast, CAAT identified them and a fourth amino acid by combining two AF2 predictions (one for each KaiB variant) with straightforward tensor operations. AF2 predictions suggested that this fourth amino acid, L64, combined with V83 were sufficient to switch the fold of KaiBRs when mutated to the corresponding amino acids in KaiBLp (E and S, respectively). NMR experiments confirmed that these two amino acid substitutions are sufficient to flip KaiBRs’s major state to the fold-switched conformation (Figure 3C). The ^1^H, ^15^N heteronuclear single quantum coherence spectrum (HSQC) of the KaiB double mutant differed dramatically from wildtype. Backbone assignments confirmed that its major state matches the minor fold-switched state of wildtype KaiB. Consistently, considerable overlap is observed between the HSQCs of the KaiB triple mutant identified previously (Figure S8).

The C-terminal domain (CTD) of the transcriptional regulator, *Escherichia coli* RfaH, was tested next. *E. coli* RfaH CTD populates a major β-roll conformation and a minor unfolded state with mixed α-helix and β-sheet character (Figure 3D) (23). This minor state may foster its ability to switch to a fully α-helical hairpin fold important for limiting transcriptional activation to specific DNA sites (24). AF2 predicts that RfaH CTD consistently assumes a fully helical hairpin fold from single sequence; though this conformation has not been observed experimentally, we use it here as a proxy for the minor state, which is structurally similar to the helical C-terminal half of the minor state. By contrast, AF2 predicts the β-roll fold for *E. coli* NusG CTD, an RfaH homolog. CAAT suggested that L142 is the most important amino acid for RfaH CTD’s structure prediction (Figure 3E). Mutating it to S (the corresponding amino acid in *E. coli* NusG’s CTD) caused AF2 to produce many (23/25) disparate, low-confidence structures, while only two good confidence structures were predicted, both of which had β-sheet folds instead of α-hairpin (Figure 3F). We interpret this shift in prediction confidence and diversity of structures to suggest that the L142S mutation destabilizes the RfaH CTD fold. Circular dichroism (CD) confirms that RfaH L142S is unfolded (Figure 3F). By contrast, H152 has a low CAAT score Figure 3E). Previous experiments indicate that H152L stabilizes RfaH’s β-roll fold (25). By contrast, AF2 predictions of H152L yield many α-hairpins with high confidence, and 80% of predicted models were predicted α-hairpin overall (Figure 3G, S7). To test the effect of the H152L mutation on RfaH CTD’s minor state, we compared its HSQC with that of wildtype. Numerous peaks corresponding to the wildtype minor state are not present in the H152L variant, suggesting the absence of a minor state on a slow exchange timescale (Figure 3G). Further, differential scanning calorimetry experiments indicate that RfaH H152L is substantially more thermostable than RfaH (64.6 and 47.4° C, respectively, Figure 3H). Thus, RfaH H152L’s lack of minor state and increased stability are not reflected in AF predictions. This predictive oversight may occur because H152 has a low CAAT score, indicating that it receives relatively little attention from AF2’s transformer.

Finally, CAAT was applied to a pair of engineered proteins with high levels of sequence identity but different folds and functions (GA and GB, Figure 3I). In this case, we used engineered variants with 77% sequence identity (GA77 and GB77, (26)) and ran CAAT with full multiple sequence alignments rather than single sequences. Structures of GA77 and GB77 are not in AF2’s training set. Bryan and Orban designed these sequences further to have 98% sequence identity but different folds and functions. CAAT reproduced their design strategy exactly (Figure 3J). In further detail, Bryan and Orban preserved the GA identities of amino acids with high CAAT scores for the GA fold and GB identities of amino acids with high CAAT scores for the GB fold. Thus, it is conceivable that applying this approach to another protein pair may suggest mutations that could switch their folds.

## Discussion

The increasing power and success of transformer-based AI models ensure their continued application to many important problems (1, 27). Nevertheless, one of their main weaknesses is lack of interpretability, which leaves hallucinations unexplained and breaks trust (8, 28, 29). By analyzing the weights of AF2, we identified some mechanistic, human-interpretable reasons for failed predictions that may extend beyond protein structure prediction. First, AF2 relies on sparse patterns of amino acids to select protein conformations. Although these patterns correspond to physically important features of proteins, they are not always sufficient to determine the fold and could be misleading, such as with XCL1. Similar associations based on incomplete information from sparse patterns may explain incorrect predictions or hallucinations in other models. Second, AF underweights the roles of amino acids outside of these sparse patterns, yet they can lead to equally drastic outcomes. Specifically, AF can be blind to the effects of mutations to low-attention amino acids. These mutations can cause obvious structural changes, however, as demonstrated by variants of XCL1, KaiB, and RfaH. Other AI models may also hyperfocus on sparse information while ignoring other important cues. This possibility is consistent with other studies showing that transformer models can become overwhelmed (30, 31).

Contrasting previous mechanistic interpretability studies on protein language models, which are more descriptive (32–34), this study converts AI interpretability into a functional tool called CAAT (Conformational Attention Analysis Tool). CAAT identifies amino acids important to both AF predictions and actual protein structure. While the one previous approach of which we are aware required hundreds of AF runs (15), CAAT achieves better results and requires only one or two AF runs (though CAAT’s performance is mildly affected by attention head extraction, Methods). Similar approaches that functionalize AI interpretability may be useful for other applications. Still, CAAT has limitations. As demonstrated, the amino acids CAAT identifies are not always sufficient to switch protein folds, and it misses other critical amino acids. Further, CAAT may not fully explain how AF selects alternative protein structures. For instance, AF-based predictions of alternative conformations can also result from overtraining or hallucinations that may not readily arise from sequence-structure relationships (35, 36). However, the two failure modes of AF2 described here may explain the tension between observations suggesting that AF2 has learned physics in some cases (37, 38) and others finding inaccuracies and absence of important conformations (11, 39, 40). The sparse patterns AF uses to predict protein structure may be enough to represent some proteins but not all. If AF could achieve a more generalized understanding of protein folding by incorporating more abstract physical concepts, such as solvent interaction, temperature, and electrostatics, its predictions may come closer to physical reality. Expanding AI architectures to apply other broad concepts may go a long way in improving models overall (41).

## Methods

### AlphaFold predictions

AlphaFold predictions were performed with ColabFold (17) version 1.5.5 for AlphaFold2 with alphafold2 weights and AlphaFold3 (5) version 3.0.0. Predictions using 5 models from both architectures were performed.

### Identification of potentially important XCL1 sites

PSI-Blast with an E-value threshold of 0.005 was used to find XCL1 homologs deposited in the PDB before April 30^th,^ 2018. The structures were grouped by structural similarity, aligned separately, and their sequence logos (42) were compared (Figure S2). Aligned amino acids with BLOSUM62 scores of ≤ −2 were then selected for testing, excluding changes to/from P and C. We tested 22R, 22F, 27F, 27T, 28E, 28I, 36K, 36L, 47R, 47L, 65L and 65R.

### XCL1 predictions

ColabFold and AF3 were initially run on the sequences of XCL1 and its homologs in single sequence mode, 3 recycles. Unlike AF2, AF3 was trained after Anc0 was added to the training set (PDB 7JH1, released 12/30/2020). Anc0 differs from other variants by a deletion and a mutation at position 28, which likely altered some of AF3’s predictions. Anc2j had the deletion but not the mutation. An N28E mutation shifted all its predictions to dimer (Figure S1B).

For the strip plots in Figure 1B, all predictions were scored using TM-align. Reference structures were 1J9O for chemokine and 2JP1 for dimer. For 2JP1 residues 1–50 were used for TM-align, ignoring predictions of a spurious C-terminal helix noted previously (16). Strip plot points were colored with the requirement that TM-scores were >0.5. If TM-scores against both reference structures were < 0.5, then the point was colored black. Only points with plDDT > 72 were plotted. The same approach was used for the AF3 strip plots in S1, but with all points were plotted regardless of plDDT.

For the scatter plots in Figure 1C, predictions were run on all variants with a C30G mutation, following the experiments from Dishman, et al. (14). Because of Anc0’s addition to AF3’s training set, the sequences of Anc2c, Anc2j, and Anc2l were also modified to contain the N28E ΔA29 mutations for AF3 runs only. A few exceptions were found in the patterns reported in Figures 1C. For both AF2 and AF3 all combinations of I,L,V resulted in dimer predictions for all variants except for I/L/V14, I/V43, L45, which produced the chemokine fold in Anc0 only (6/17965 models). For AF2, charged residues in positions 14, 43, and 48 caused some unphysical dimer predictions in variants with sequences closer to XCL1 when K, T, H, N were in position 14 or N was in position 2. Removing these cases, 25 dimer predictions remained out of 17,965. All had S or T in positions 43 or 48. These predictions, which represent 0.2% of predictions are not shown in Figure 1C. Predictions were generated by making all I,L,V combinations in positions 14, 43, 48, all 5 AF2 models, 3 random seeds. Charged residue predictions were made by randomly sampling 1000 combinations of amino acids in positions 14, 43, and 48 except for the patterns mentioned about, all 5 AF2 models, 3 random seeds. For AF3, the same chemokine prediction for Anc0 was observed for I/L/V14, I/V43, L45 as in AF2; these points, representing < 1% of all dimer predictions, were not shown in Figure 1C. Otherwise, no exceptional sequence rules were observed for AF3 predictions. Figure 1C shows all AF3 predictions with plDDT scores ≥ 62 and AF2 predictions with plDDT scores ≥ 70. The same trend holds for AF3 predictions with plDDT scores ≥ 70, but few dimer predictions were made with predictions above this threshold.

### Attention heads

Attention heads were extracted from each run of ColabFold (all 5 models, 3 recycles) by deactivating AF’s custom memory optimization, allowing entire attention heads to be saved chronologically from the GPU, via the jax.debug module, as the model gets executed. This approach causes a slowdown in the runtime of AF, but we believe this is the optimal tradeoff between simplicity and efficiency that does not significantly impact the utility of CAAT. There are two primary sources of slowdown. The first is due to turning off AF’s memory optimization, which breaks each attention head into smaller pieces for enhanced parallelization. We remove this optimization because it obfuscates the location of each attention head in the model, inhibiting the interpretability of our results. The second source of slowdown comes from the time cost of transferring attention head values from the GPU to the CPU during execution. For different computational experiments with greater memory requirements, more sophisticated memory management may be required.

### Attention analysis

Attention heads with shape nx4xnxn were selected from all attention heads. Axes 0, 1, and 2 were summed then averaged. Min-max normalization was performed, and the resulting scaled average was plotted (Figure 2C). The 5 amino acids with the highest average attention were mutated to Alanine. 100 5-amino acid mutations were also made to Anc0 and XCL1. TM-align was run with their respective PDB IDs (7JH1 and 2JP1) and plotted as box and whisker plots (Figure 2C). Anc0 and XCL1 sequences were aligned and BLOSUM62 scores were obtained for all aligned amino acids. BLOSUM62 score was multiplied by the average attention previously obtained. Negative scores were plotted to obtain the amino acids important to XCL1 and Anc0 in Figure 2D. Attention values were systematically lower at N- and C- termini than in the center of the protein. To avoid false positives in CAAT in cases where aligned sequences had gaps at termini, attention differences between the first/last 5 amino acids after/before a terminal gap were ignored.

### XCL1 experiments

Human XCL1(1–72) protein, which lacks the disordered C-terminal tail (residues 73–93), was expressed in DL39(DE3)E. coli, using a chemically defined media supplemented with 100 mg/L of 4-fluorophenylalanine and all 19 other naturally occurring amino acids as described. [REF PMID 27414758]. The inclusion body pellet containing hXCL1(1–72) was refolded and purified as previously described [REF PMID 25497737]. Incorporation of a single fluorine atom was verified by electrospray mass spectrometry and presumed to reflect the substitution of F39, the only phenylalanine in the human XCL1 sequence.^19^F NMR spectra of 4-F-Phe labeled hXCL1(1–72) (100 μM) in aqueous buffer containing 20 mM NaPO4, 50 mM NaCl, pH 6.5 were acquired on a Bruker Avance III 500 NMR spectrometer at 35 °C using a spectral width of 37500 Hz, 256 scans, and 8192 total data points. Trifluoroacetic acid (100 μM) was added to each sample and used as an internal standard for normalization of peak intensities. Chemical shifts referencing was to an external trichloro-fluoromethane (0 ^19^F ppm).

### Protein expression, purification

The RfaH C-terminal Domain (24) was ordered from Twist Biosciences (San Francisco, CA) and cloned into a pPal7 vector with an N-terminal 6x his-tag and bdSUMO solubility tag (43). H152L and L142S variants were generated using site-directed mutagenesis and confirmed by whole-plasmid sequencing. ecNusG CTD, KaiB RS and all described variants were ordered from Genscript (Piscataway, NJ) and cloned into pET28a vectors with an N-terminal 14x his-tag and bdSUMO solubility tag. All plasmids were transformed into *E. coli* BL21 (DE3) cells (Agilent).

### RfaH and NusG

[^15^N]-enriched protein expression was based on previous methods (44, 45). 2.8L flasks containing 1 L of Terrific Broth (Research Products International) were autoclaved. From a Luria Broth(LB)/Agar plate + 100 μg/mL ampicillin or frozen cell stock, a 25 mL Terrific Broth + 100 μg/mL ampicillin or 50 μg/mL kanamycin overnight culture was inoculated and grown at 37 °C. The next day, 1 L Terrific Broth flasks + 100 μg/mL ampicillin or 50 μg/mL kanamycin were inoculated with 2.5 mL overnight culture. At OD_600_ = 0.6, flasks were spun at 6,000 rcf, 4 °C, for 15 minutes. Supernatant was poured off and cells resuspended in half of the original volume of 1X M9 salts, pH 7.4. Cells were again pelleted at 6,000 rcf, 4 °C, for 15 minutes, and then resuspended in 1/4 the original volume, in sterile filtered 2X M9 salts, 4 g/L D-glucose, 1 g/L ^15^NH_4_Cl, 1X Modified Eagles medium, 2 mM MgSO_4_, 100 μg/mL ampicillin or 50 μg/mL kanamycin, 5 mL of 200 X trace element solution (46). Unenriched protein was prepared in a similar fashion, except the overnight culture was used to inoculate 0.5 or 1 L Terrific Broth media + 100 μg/mL ampicillin or 50 μg/mL kanamycin and shaken at 180 rpm at 37 °C to an OD_600_ = 0.6. Cells were transferred to a shaker at 18 °C, and shaken for 30 minutes, after which, Isopropyl β-D-1-thiogalactopyranoside (IPTG) was added to a final concentration of 200 μM.

The next day, cells were harvested by centrifuging at 6,000 rcf, 4 °C, for 15 minutes. Cells were resuspended in 50 mM tris(hydroxymethyl)aminomethane (tris), 150 mM NaCl, 20 mM imidazole, 1 mM dithiothreitol, 5% glycerol, pH 8.8 at 4 °C. ½ cOmplete protease inhibitor tablet (Roche) was added, along with 100 U Benzonase Nuclease (Millipore). Cells were lysed (Microfluidics, M110P), spun at 38,724 rcf, 4 °C for 45 minutes, and then syringe filtered (0.45 μm).

Filtered lysate was then loaded on HisTrap HP (Cytiva) column, washed with 50 mM KP_i_, 100 mM NaCl, pH = 7.4, then eluted from the column with a 4 CV linear gradient to 20% elution buffer (50 mM KP_i_, 100 mM NaCl, 500 mM imidazole, pH 7.4), followed by immediate elution 100% elution buffer. Fractions containing protein of interest were pooled and 100 ng bdSUMO protease was added, and the sample dialyzed overnight at 4° C in wash buffer with 0.2 mM tris(2-carboxyethyl)phosphine) (TCEP) added. The next day, the cleaved sample was loaded again on the HisTrap column and washed, collecting the flow-through. The flow-though was concentrated and then further purified using size exclusion chromatography, pre-equilibrated in 100 mM KP_i_, pH 7.4 (HiLoad Superdex 75 pg, Cytiva). Pure fractions were determined by SDS-page gel analysis, pooled, and purity was validated by mass spectrometry (6230 ESI-TOF LC/MS, Agilent). Protein concentrations assessed using absorbance at 205 nm (47).

### KaiB Rs and variants

[^15^N]-enriched samples were expressed in the same fashion as described above. Uniformly [^2^H/^13^C/^15^N]-enriched KaiB RS L64E;V83S was prepared as follows. Multiple colonies were picked and grown in 3 mL LB media (Research Products International) dissolved in 99.8% D_2_O (Cambridge Isotope Laboratories) and grown overnight at 37 °C. The next day, The 2.5 mL overnight was diluted into 22.5 mL modified M9+ medium(48) with 3 g/L [^2^H,^13^C] glucose and 1.5 g/L ^15^NH_4_Cl in 99.8% D_2_O and grown for 24 hours at 37 °C. The next day, the culture was diluted to 1 L modified M9+ and at OD_600_= 0.8, the culture was induced with 0.3 mM IPTG in D_2_O. After 7 hours of expression at 37 °C, the cells were harvested as described above.

Cells were resuspended, lysed, underwent nickel affinity purification, SUMO cleavage, and secondary nickel affinity purification as described above, except TCEP was added to nickel affinity buffers. After, the sample was diluted 5-fold in 20 mM MES, 0.5 mM TCEP, pH = 5.5, and then purified via cation-affinity chromatography using a home-made SP-resin column (Cytiva). The protein was isolated over a 5 CV linear gradient from 0–40% 20 mM MES, 2 M NaCl, 0.5 mM TCEP, pH = 5.5. Pure fractions were determined by SDS-page gel analysis, pooled, and purity was validated by mass spectrometry. Protein concentrations assessed using absorbance at 205 nm (47).

### NMR Spectroscopy

NMR measurements were acquired on Bruker Avance II 600 MHz, Neo 600 MHz, or Avance II 800 MHz spectrometers equipped with z-gradient cryogenic probes. Spectra were processed using NMRpipe (49). RfaH CTD and CTD H152L measurements made in 20 mM potassium phosphate, 100 mM NaCl, 1 mM ethylenediaminetetraacetic acid, 10% (v/v) D_2_O, pH 6.5 at 23 °C. KaiB measurements were made in 25 mM MOPS, 50 mM NaCl, 2 mM TCEP, 1 mM PMSF, 5% D_2_O, pH = 6.5 at 35 °C. Backbone assignments of KaiB L64E;V83S were performed at 600 μM, 35 °C using TROSY-HSQC, HNCO, HNCA, HNCACB, HN(CO)CA, HN(CO)CACB experiments. Non-uniform sampled data was reconstructed using SMILE (50) and all data processed using NMRPipe. Assignments performed using CCPN (51). TALOS+ was used for secondary structure prediction (52).

### Circular dichroism measurements

Measurements were made on a Chirascan Q100 (Applied Photophysics) in 1 mm quartz cuvettes (Hellma) in 100 mM Potassium Phosphate, pH 7.4. Concentration of RfaH CTD L142S was 17.3 μM, and the concentrations of RfaH CTD, RfaH CTD H152L, ecNusG CTD were 20 μM. Data was collected in 1 nm step sizes, 1 nm/s scan rate, at 20 °C. 10 scans were made for each protein, with measurements averaged. Triplicated buffer blanks were averaged and subtracted. Spectra were then converted to Molar Residue Elipicity [*θ*]_*MRE*_ using equation 1:

$${\left[\theta \right]}_{MRE}=\frac{\theta *\varepsilon }{L*N*A}$$

Where *θ* is the measured ellipticity, *ε* is the molar absorbtivity at 205 nm estimated from https://spin.niddk.nih.gov/clore/Software/A205.html (47), L is the cuvette pathlength, N is the number of amino acids, and A is the measured absorbance at 205 nm (Nanodrop One, Thermo Scientific).

### Differential scanning calorimetry measurements

RfaH CTD and RfaH CTD H152L were dialyzed into 4 L 10 mM potassium phosphate, pH = 7.4 for 48 hours. The dialysis buffer was sterile filtered and used to blank the PEAQ-DSC (Malvern Panalytical). Measurements were made at a 60 °C/hour scan rate from 10 °C to 95 °C. Data was fit using MicroCal PEAKQ-DSC Software version 1.64, using the two-state *ΔC*_p_≠0 fitting model. Reported errors are derived from the fit.

## Supplementary Material

Supplement 1

## Data Availability

Code and data can be found at: https://github.com/prameshsharma25/CAAT
