# Explaining how mutations affect AlphaFold predictions

**DOI:** 10.64898/2025.12.30.697132

**Published:** 2026-01-13

**Authors:** Madeleine F. Clore, Joseph F. Thole, Suchetan Dontha, Pramesh Sharma, Naomi Greenberg, Marie-Paule Strub, Mary Starich, Davin Jensen, Brian F. Volkman, Matthew Coudron, Lauren L. Porter

## Abstract

Transformer models, neural networks that learn context by identifying relationships in sequential data, underpin many recent advances in artificial intelligence. Nevertheless, their inner workings are difficult to explain. Here, we find that a transformer model within the AlphaFold architecture uses simple, sparse patterns of amino acids to select protein conformations. To identify these patterns, we developed a straightforward algorithm called Conformational Attention Analysis Tool (CAAT). CAAT identifies amino acid positions that affect AlphaFold’s predictions substantially when modified. These effects are corroborated by experiments in several cases. By contrast, modifying amino acids ignored by CAAT affects AlphaFold predictions less, regardless of experimental ground truth. Our results demonstrate that CAAT successfully identifies the positions of some amino acids important for protein structure prediction, narrowing the search space required to predict effective mutations and suggesting a framework that can be applied to other transformer-based neural networks.

## Full Text Availability

The license terms selected by the author(s) for this preprint version do not permit archiving in PMC. The full text is available from the preprint server.

