# Supplementary material for: Explaining how mutations affect AlphaFold predictions
Source: bioRxiv. 2025 Dec 31:2025.12.30.697132. Preprint. [Version 1] doi: 10.64898/2025.12.30.697132 (PMC12776252; doi:10.64898/2025.12.30.697132)
Supplement: Supplementary file 1 [file media-1.pdf]

Supplementary information for

Explaining how mutations affect AlphaFold predictions

Madeleine F. Clore[1,†], Joseph F. Thole[1,2,†], Suchetan Dontha[3], Pramesh Sharma[1], Davin Jensen[4], Brian F. Volkman[4], Matthew Coudron[3], and Lauren L. Porter[1,2,*]

1. National Library of Medicine, National Institutes of Health, Bethesda MD 20894, USA

2. National Heart Lung and Blood Institute, National Institutes of Health, Bethesda MD, 20894, USA

3. The Joint Center for Quantum Information and Computer Science, University of Maryland, College Park, MD 20742

4. Department of Biochemistry, Medical College of Wisconsin, Milwaukee, WI 53226, USA

[†]Contributed equally

*Correspondence - porterll@nih.gov

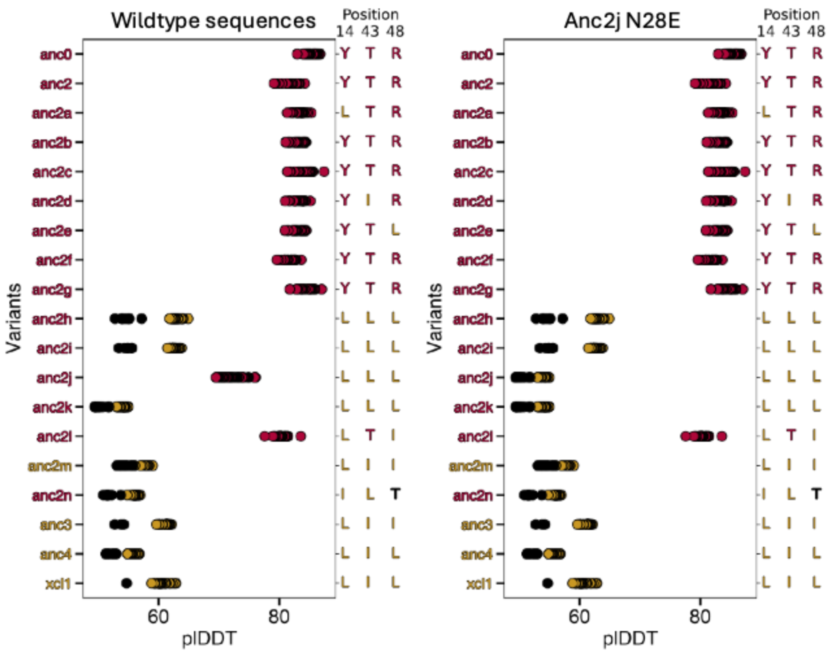

Figure S1. AlphaFold3 also overpredicts the XCL1 dimer conformation. A. Anc2h, 2i, 2k, and 2n are predicted to assume the dimer conformation (yellow circles) though NMR experiments do not detect it. Red circles are chemokine predictions; black are unfolded. B. Unlike AF2, AF3 was trained after Anc0 was added to the training set (PDB 7JH1, released 12/30/2020). Anc0 differs from other variants by a deletion and a mutation at position 28. Anc2j had the deletion but not the mutation. An N28E mutation shifted all of its predictions to dimer.

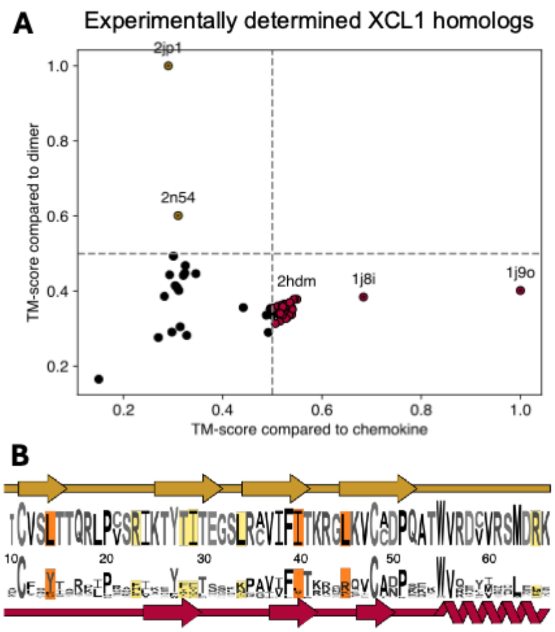

Figure S2. Identifying different sequence properties of fold-switching and single-folding XCL1 homologs. A. PDB entries homologous to the XCL1 sequence were identified through protein BLAST and their folds were characterized. Of the entries identified, 42 assumed the chemokine fold (red dots in bottom right quadrant) and 2 assumed the dimer fold (yellow dots in top left quadrant). Other entries (black dots) were not included. B. Sequence logos of the 2 dimer homologs (top) and 42 chemokine homologs (bottom). Differing positions highlighted in yellow; positions 14, 43, and 48 (numbered 14, 40, and 45 because the alignment lacks gaps) are orange.

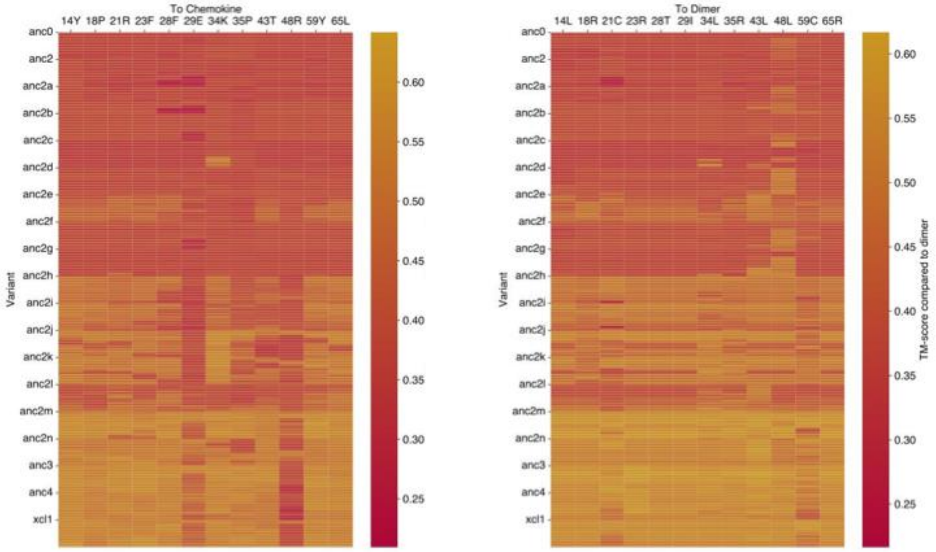

Figure S3. Point mutations have minimal effect on AF2 predictions of XCL1 variant structures. Positions differing in sequence logos (Figure S2B) with a BLOSUM62 score of -2 or more were mutated both ways (chemokine, left; dimer, right). Most mutations had minimal effects on overall predictions; mutations to position 48 had the strongest effect both ways.

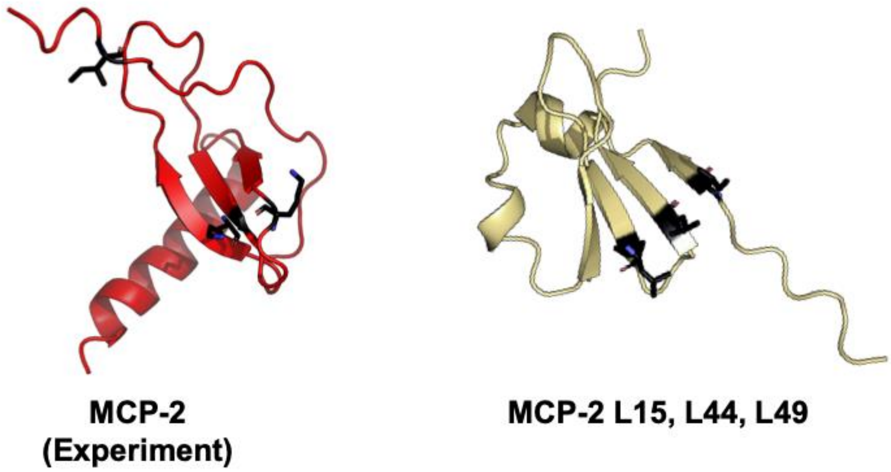

Figure S4. AF2 predicts that mutations to L in the same 3 positions cause MCP-2 to assume the dimer fold. This is unlikely since it is outside of the XCL1 clade. PDB ID of experimentally determined MCP-2: 1ESR.

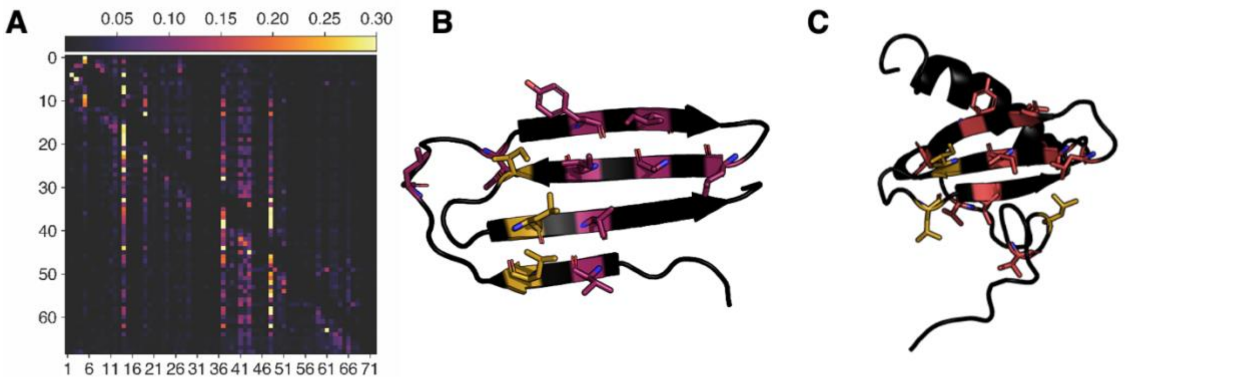

Figure S5. AF2 pays attention to the dimer interaction network when predicting the dimer fold. A. Attended amino acids correspond to vertical lines in the attention head. B. The attended amino acids in A are mapped onto XCL1's dimer structure and correspond to an interaction network; positions 14, 43, and 48 are highlighted in gold; the other attended positions are purple. C. The interaction network in the XCL1 attention head and in the dimer fold is not present in the chemokine fold; positions 14, 43, and 48 are highlighted in gold; the other attended positions are orange.

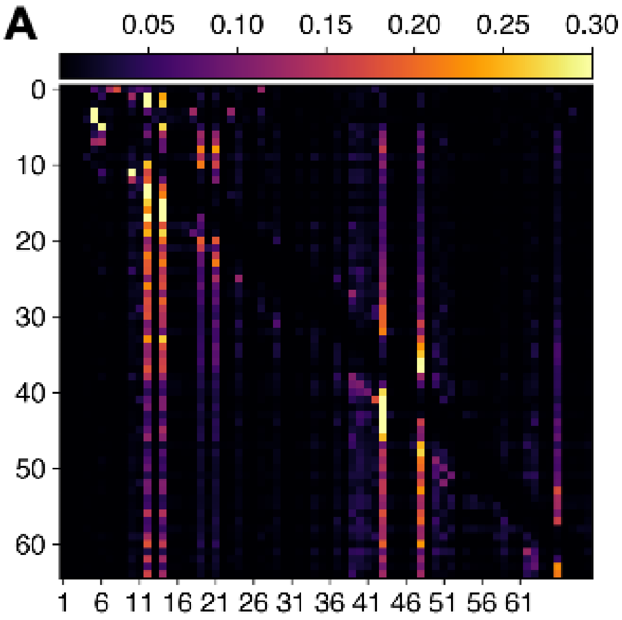

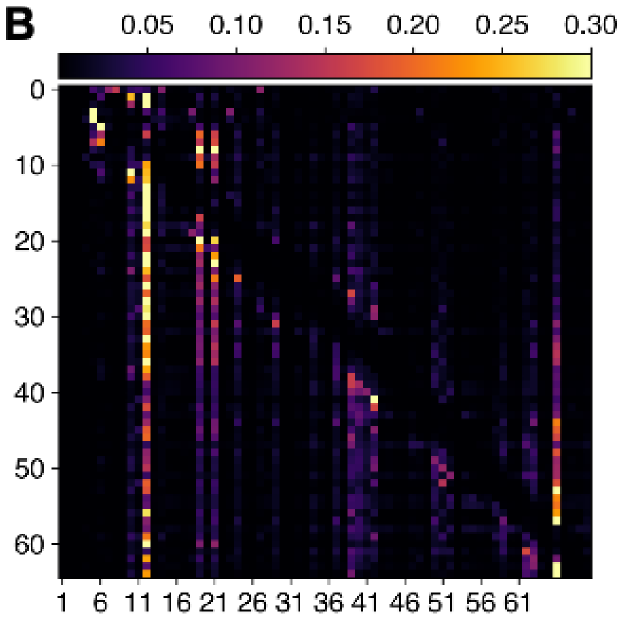

Figure S6. AF2 attention patterns for Anc2f L14, L43, and L48 and wildtype Anc2f resemble those of XCL1 and Anc0, respectively. A. The attention pattern of Anc2f L14, L43, L48 corresponds to an interaction network unique to the XCL1 dimer fold. Note that positions 14, 43, and 48 are lit up as they are for XCL1 in Figure 2B. AF2 predicts that Anc2f L14, L43, L48 assumes the dimer fold. B. The attention pattern of wildtype Anc2f does not correspond to the interaction network unique to the XCL1 dimer fold. Note that positions 14, 43, and 48 are not lit up as they are for XCL1 in Figure 2B; rather the attention pattern of Anc2f resembles Anc0. AF2 predicts that Anc2f assumes the chemokine fold. Attention patterns were taken at residue 0, head 0, layer 6, recycle 0 as in Figure 2.

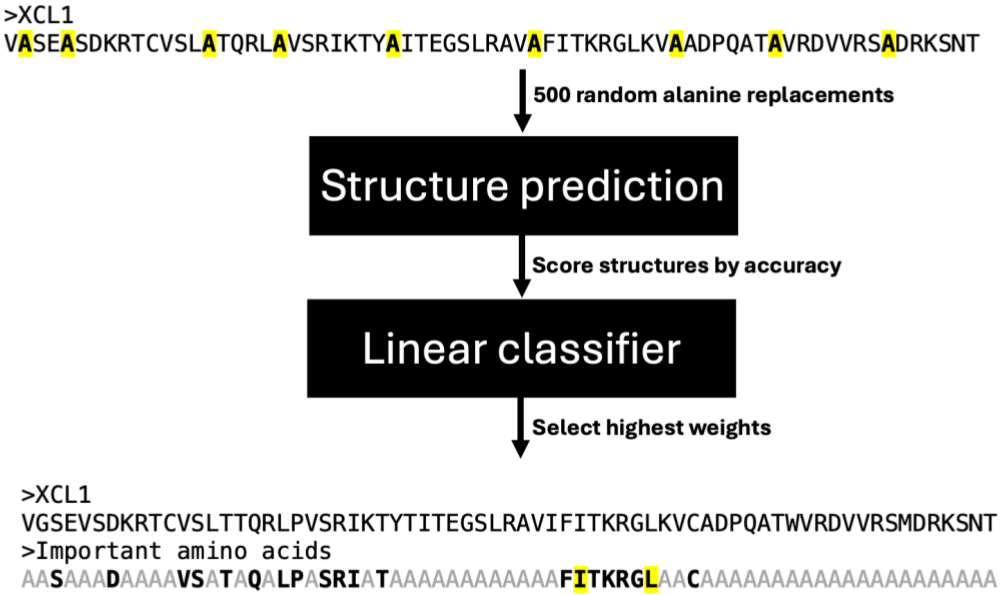

Figure S7. Linear classifier approach to finding important residues for XCL1 folding. Ten percent of the amino acids in the XCL1 sequence were mutated to alanine at random, resulting in 500 different variants. AF2 was run on each variant, all 5 models, 5 random seeds, 3 recycles. The TM-scores of each resulting prediction (25 for each variant) were quantified, and the overall score for a given set of mutations was quantified as Average(TM-score)-Standard deviation(TM-score) over all 25 models. This approach rewarded consistency in predictions, making it more likely to identify amino acids important to all 5 AF2 models. The top 30% most important amino acids identified by a linear classifier (indicated by largest positive weights) are highlighted in >*Important amino acids* sequence. Positions 43 and 48 are highlighted, and the linear classifier identified position 48 as most important overall. Position 14 was not identified as important because the dimer conformation was predicted without trouble in response to alanine mutation. Mutating to a different amino acid, such as tyrosine, may have a different effect.

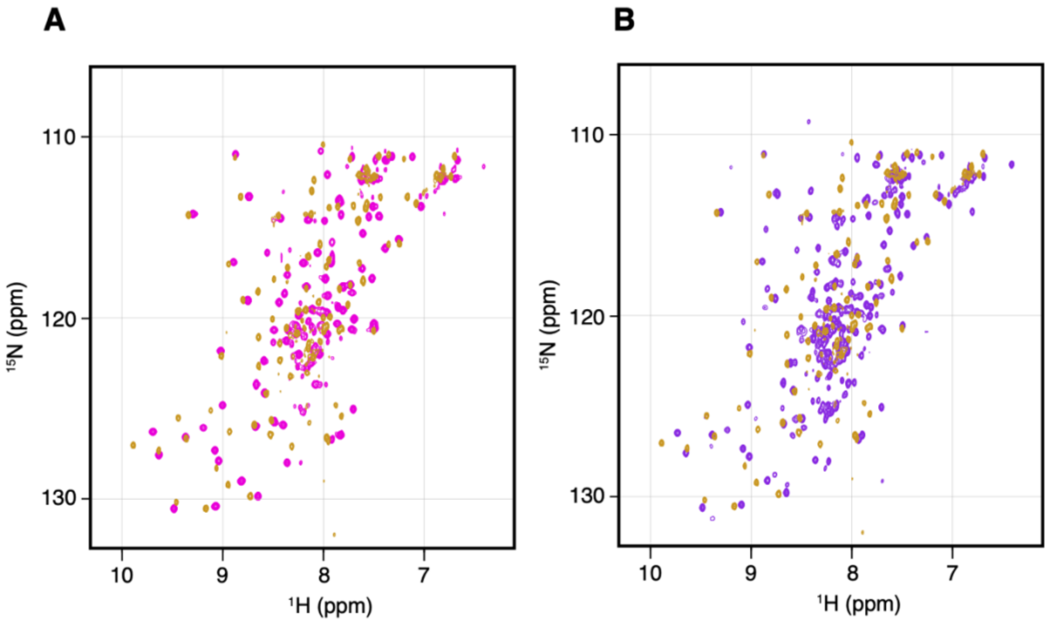

Figure S8. KaiBRs L64E, V83S has an HSQC similar to KaiBRs I67R, V83D, N64A, which was shown to assume the fold-switched conformation previously. A. HSQC overlays of KaiBRs L64E, V83S (gold) with KaiBRs I67R, V83D, N84A (pink). B. AlphaFold also predicts that KaiBRs I67R, V83D assumes the fold-switched form, but with lower confidence. Its HSQC (purple), which shows more broad peaks, is consistent with a protein that is not as well folded as KaiBRs L64E, V83S (gold).
